# Distinct inactivated bacterial-based immune modulators vary in their therapeutic efficacies for treating disease based on the organ site of pathology

**DOI:** 10.1038/s41598-020-62735-z

**Published:** 2020-04-03

**Authors:** Shirin Kalyan, Mark Bazett, Ho Pan Sham, Momir Bosiljcic, Beryl Luk, Salim Dhanji, Amanda M. Costa, Stephanie W. Y. Wong, Mihai G. Netea, David W. Mullins, Hal Gunn

**Affiliations:** 1 Qu Biologics Inc., Burnaby, BC V5G 4X4 Canada; 2 Department of Medicine, University of British Columbia, Vancouver, Canada; 3 Department of Internal Medicine, Radboud University Medical Center, Nijmegen, Netherlands; 4 Department of Microbiology and Immunology and Department of Medical Education, Geisel School of Medicine at Dartmouth, Hanover, NH 03755 USA

**Keywords:** Immunology, Immunization

## Abstract

Recent developments in understanding how the functional phenotype of the innate immune system is programmed has led to paradigm-shifting views on immunomodulation. These advances have overturned two long-held dogmas: (1) only adaptive immunity confers immunological memory; and, (2) innate immunity lacks specificity. This work describes the observation that innate immune effector cells appear to be differentially recruited to specific pathological sites when mobilized by distinct inactivated bacterial-based stimuli administered subcutaneously. The studies presented suggest that the immune system, upon detecting the first signs of a potential infection by a specific pathogen, tends to direct its resources to the compartment from which that pathogen is most likely originating. The findings from this work puts forth the novel hypothesis that the immunotherapeutic efficacy of a microbial-based stimulus for innate immune mobilization depends on the correct selection of the microbial species used as the stimulant and its relationship to the organ in which the pathology is present.

## Introduction

In 1899, cancer researcher D’Arcy Power noted: “Where malaria is common, cancer is rare.”^1^ For centuries, physicians have observed that acute infection can be associated with spontaneous cancer regression, and it is now well appreciated that the potent immune response to the threat of acute infection can overcome malignancy^2,3^. One of the best known early clinical applications of this observation was developed in the 1890’s by Dr. William Coley who used an inactivated bacterial cocktail, which came to be known as Coley’s Toxin, to treat various types of cancers and managed to achieve durable remission in many cases^2^. Coley believed that simulation of a naturally occurring acute infection was essential for efficacy of his approach, and patients were injected every day or every other day directly into the primary tumour, when accessible, for at least six months^2^. At least 15 epidemiological studies have examined the possible link between infectious disease and cancer, with all but one supporting an association between infection and reduced incidence of neoplasms^2^. Yet, intra-vesical administration of Bacillus Galmette-Guerin (BCG) for the treatment of high-risk non-muscle invasive bladder cancer is the only microbe-based therapy that is currently part of the approved standard of care^4^. The failure to realize the full potential of this immunotherapeutic approach can be attributed to a number of factors, including lack of sufficient characterization of the immunological mechanism(s) driving the therapeutic outcome and difficulties with obtaining consistent results. However, recent pivotal developments in understanding the basis of what is now coined trained innate immunity, which encompasses changes in the programming of the innate immune response through experience, have far-reaching potential to improve our understanding of the anti-cancer immunological response induced by acute infection^5,6^. This new deeper appreciation of innate immune system adaptation and reprogramming is projected to meaningfully advance the use of microbial-based treatments for not only cancer, but also for many of the increasingly prevalent immune-based diseases we now contend with, including inflammatory bowel disease, allergies and autoimmune disorders^5–7^.

We recently characterized the key immune cellular and molecular pathways that were activated by using a microbe-based stimulus that mimicked an acute infection and led to the marked reduction in tumour burden in two different lung cancer models^8^. The treatment used was formulated from an inactivated lung pathogen, a derivative of a clinical isolate of *Klebsiella*, which is subcutaneously administered every second day^8^. Efficacy in reducing lung tumour burden was shown to be (1) dependent on the animals having had previous exposure to *Klebsiella* and (2) independent of adaptive immunity^8^. In the work presented here, we describe the novel observation that there appears to be organ-specific recruitment of bone-marrow derived innate immune cells and disease amelioration that is regulated by the identity of the inactivated bacteria used to formulate the subcutaneously administered treatment. Table 1 lists the microbial-based formulations used for these studies and their origin. Our data suggest that it may be possible to therapeutically target different organ sites of pathology by administering a microbial stimulus that is derived from a bacterial species that is endogenous to that organ niche.

**Table 1:** Microbial-based treatments tested and their origin.

Microbial Treatment	Bacterial Species and Their Characteristics	Source
QBKPN	*Klebsiella variicola:* Gram-negative, rod-shaped, non-motile bacteria closely related to *Klebsiella pneumoniae*	Clinical isolate from the lungs of a patient with pneumonia
QBECO	*Escherichia coli:* Gram-negative, rod-shaped coliform bacteria, which functions as a facultative anaerobe	Clinical isolate from a patient with bloody diarrhea
QBSAU	*Staphylococcus aureus:* Gram-positive, round-shaped bacteria that is non-motile and a facultative anaerobe	Clinical isolate from a patient with a subcutaneous abscess

## Results

### Microbial-based immunotherapy agents demonstrate anti-cancer efficacy in an organ-specific manner

**Figure 1:**
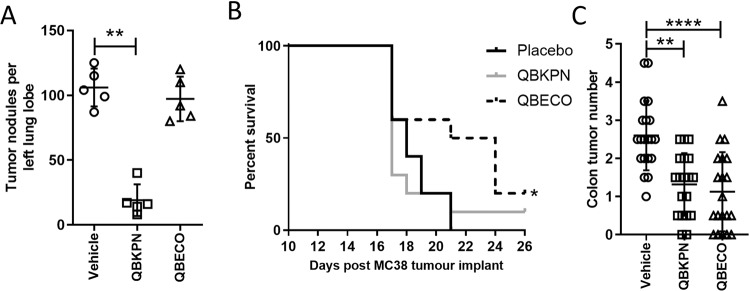
Microbial-based therapies, QBKPN and QBECO, have different organ-specific anti-cancer efficacies. (**A**) Lung tumour count in a lung cancer model; lungs of C57BL/6 mice were seeded with Lewis Lung Carcinoma (LLC)-red fluorescent protein (RFP) cells by tail vein injection and treated with vehicle, QBKPN, or QBECO. N = 5 mice per group. Left lung tumour nodule counts were completed 15 days after tumor cell inoculation. (**B**) Survival in a gastrointestinal cancer model; intraperitoneal space of C57BL/6 mice were seeded with MC-38 adenocarcinoma cells by intraperitoneal injection and treated with vehicle, QBKPN, or QBECO. Survival curves to day 26 after tumour seeding. N = 10 mice per group. (**C**) Tumour counts in a spontaneous colon cancer model using C57BL/6 mice; cancer in the colon was induced with AOM/DSS exposure, and mice were treated with vehicle, QBECO or QBKPN. Tumour counts were performed at day 70 post-AOM treatment. N = 19–20 mice per group. For each experiment, mice were given the designated treatment by subcutaneous injection every second day starting 10 days before tumour seeding and continuing throughout the experiment. Data is shown with the mean ± SD. *p < 0.05; **p < 0.01; ****p < 0.0001, Kruskal-Wallis test with Dunn’s multiple comparisons and Log-rank test (survival analysis).

A subcutaneously injected microbial-based immunotherapy made from an inactivated strain of *Klebsiella*, called QBKPN, was previously tested in a Lewis Lung Carcinoma model^8^. It was observed that mice without prior lung exposure to *Klebsiella* did not experience a reduction in tumour burden with QBKPN treatment, whereas mice with prior exposure to *Klebsiella* did^8^. Here we show that when we administer a similarly formulated immunotherapy based on *Escherichia coli*, called QBECO, there is little relative efficacy in reducing lung cancer burden in the Lewis Lung Carcinoma model, despite all animals likely having exposure to *E. coli* (Fig. 1A). We replicated the previous findings in a different lung cancer model using B16F10 melanoma cells (Supplemental Fig. 1A), which suggests QBKPN’s anti-cancer efficacy is not cancer cell type specific, rather it appears to be applicable to different cancer cell types growing in the lungs. We hypothesized that QBECO may have a therapeutic anti-tumour effect in a compartment likely to be exposed to *E. coli*. We first used an intraperitoneal cancer model since *E. coli* infection is the most common cause of Gram-negative peritonitis^9^. MC-38 adenocarcinoma cells were administered by intraperitoneal injection to establish the cancer^10^. In this model every second day subcutaneous administration of QBECO significantly increased survival compared to vehicle treatment; whereas, QBKPN treatment was less efficacious (Fig. 1B). QBECO treatment also improved survival when pancreatic cancer cells were seeded intraperitoneally (Supplemental Fig. 1B). Thus, QBECO treatment improved survival in animals with cancer in the peritoneal region independent of cancer cell type, corroborating the analogous observation with respect to QBKPN’s anti-cancer efficacy in the lungs. Collectively, these findings suggest there are organ-specific immune effects of these microbial treatments and that these subcutaneously administered products are sampled by immune cells in the targeted organ niche. To exclude the possibility that the efficacy of these microbial-based treatments was a result solely of reduced seeding of injected cancer cells, we tested both QBKPN and QBECO in a chemically-induced colon cancer model in which cancer develops with exposure to DSS/AOM over a 70 day period^11,12^. Because both *Klebsiella* species and *E. coli* are enteric bacteria in mice, both QBKPN and QBECO treatment succeeded in reducing tumour burden in the colon compared to vehicle-treated mice in this model (Fig. 1C). A biodistribution study using *in vivo* imaging of fluorescently labelled QBKPN (Cy5.5-QBKPN) confirmed that the product is systemically distributed (Supplemental Fig. 2A); peak levels in blood were detected at 2 hours after administration and dropped subsequently (Supplemental Fig. 2B).

### Characterizing the lung-specific immune response induced by QBKPN compared to QBECO in mice with lung cancer

**Figure 2:**
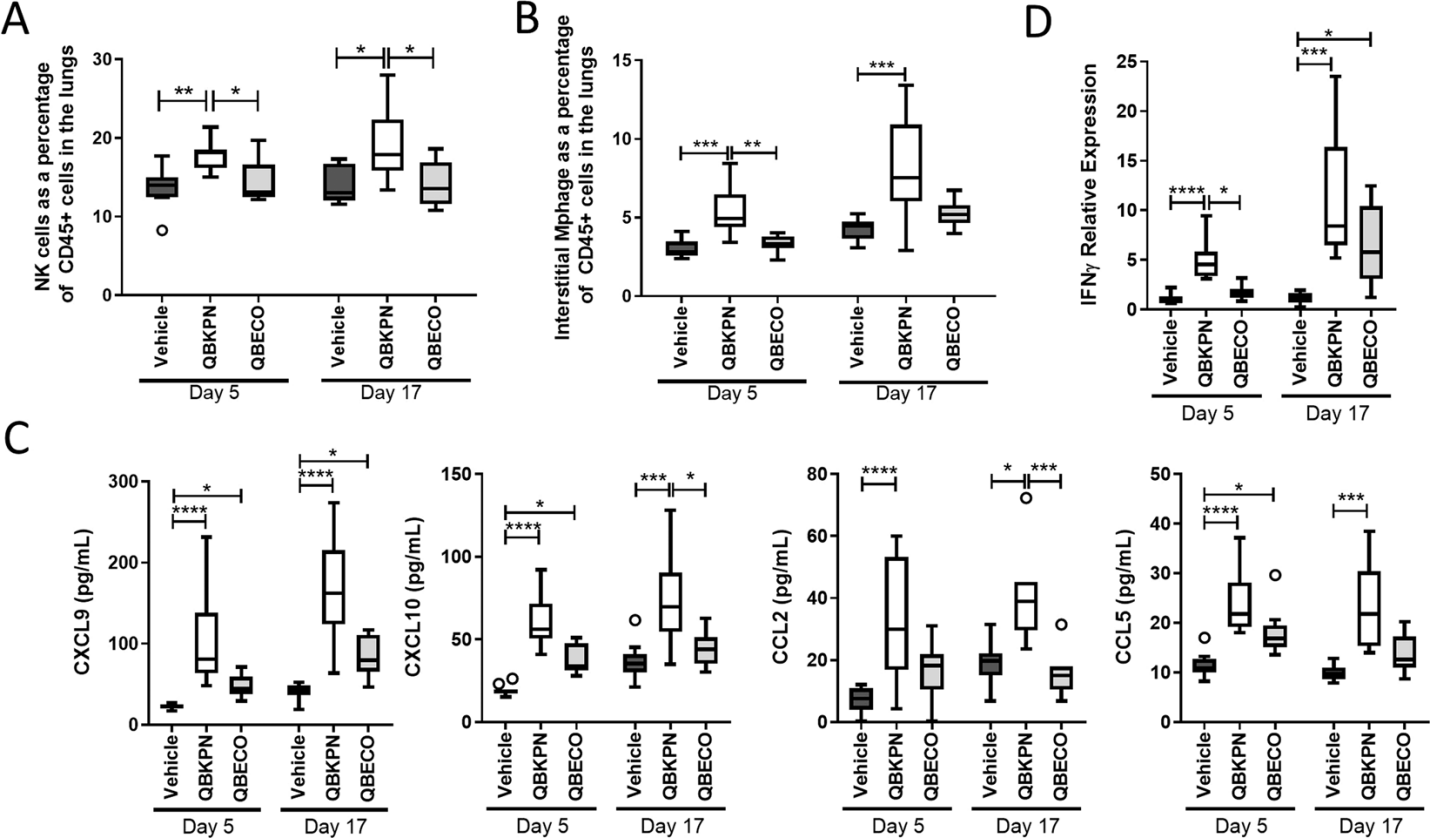
QBKPN, but not QBECO, induces lung specific immune changes in a lung cancer model. (**A,B**) Lung immunophenotyping of C57BL/6 mice in the B16F10 lung cancer model at 5 and 17 days post tumour inoculation for **(A)** NK cells (NK1.1^+^CD3^−^) and **(B)** interstitial macrophages (CD11b^+^CD24^−^CD64^+^Ly6G^−^) as a percentage of CD45^+^ cells. Data represented as Tukey boxplots; n = 9–10 mice per group. **(C)** Protein levels in lung homogenates of CXCL9, CXCL10, CCL2 and CCL5 in the B16F10 lung cancer model at 5 and 17 days post tumour inoculation. Cytokines were measured by 31-cytoplex; additional measured cytokines are in Supplemental Fig. 3. Data represented as Tukey boxplots. N = 8–10 mice per group. **(D)** IFNγ expression in the lungs tissue in the B16F10 lung cancer model at 5 and 17 days post tumour inoculation as measured by qRT-PCR. Data presented using Tukey boxplots; n = 9–10 mice per group. In each experiment, mice were treated with vehicle, QBKPN, or QBECO by subcutaneous injections every second day starting 10 days before tumour seeding and continuing throughout the experiment. *p < 0.05; **p < 0.01; ***p < 0.001; ****p < 0.0001, Kruskal-Wallis test with Dunn’s multiple comparisons.

Immuno-phenotyping was used to characterize the lung specific immune changes induced by QBKPN compared to QBECO treatment using the murine B16F10 lung cancer model. QBKPN administration increased the percentage of NK cells and interstitial macrophages in the lungs when assessed at both days 5 and 17 after tumour inoculation (corresponding to 15 and 27 days of QBKPN treatment, respectively). In contrast, QBECO treatment did not change the proportion of either NK cells or interstitial macrophages in the lungs relative to vehicle treated control mice (Fig. 2A,B).

**Figure 3:**
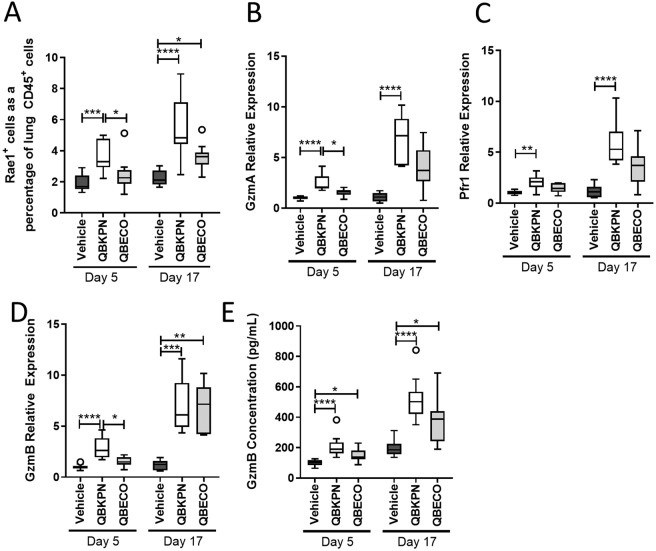
QBKPN, but not QBECO, activates anti-cancer mechanism in lungs. (**A**) Rae1^+^ expression on CD45^+^ cells in the lungs of C57Bl/6 mice in the B16F10 lung cancer model at 5 and 17 days post tumour inoculation, as assessed by flow cytometry. **(B–D)** Gene expression in the lungs of B16F10 cancer bearing mice at 5 and 17 days for **(B)** Granzyme A, **(C)** Perforin 1, and **(D)** Granzyme B. **(E)** Protein level of Granzyme B as measured by ELISA in the lungs of mice in the B16F10 lung cancer model at 5 and 17 days post tumour inoculation. For all studies, data presented using Tukey boxplots. N = 9–10 mice per group. In each experiment, mice were treated with vehicle, QBKPN, or QBECO by subcutaneous injections every second day starting 10 days before tumour seeding and continuing throughout the experiment. *p < 0.05; **p < 0.01; ***p < 0.001; ****p < 0.0001, Kruskal-Wallis test with Dunn’s multiple comparisons.

To account for the greater recruitment of NK cells and macrophages to the lungs by QBKPN vs. QBECO treatment, lung chemokine concentrations were quantified. Relative to both vehicle control and QBECO, treatment with QBKPN led to higher levels of chemokines important for the recruitment and activation of NK cells and macrophages, including CCL2, CCL5, and IFNγ-inducible chemokines, CXCL9 and CXCL10, at both 5 and 17 days following tumour inoculation (Fig. 2C). IFNγ gene expression was increased markedly increased with QBKPN treatment compared to QBECO at the early (day 5) timepoint measured (Fig. 2D). This is in line with the observation that QBKPN treatment turned on the local innate lung immune response in a manner similar to that of an acute infection, as evidenced by an increase in local granulocyte colony stimulating factor (G-CSF) production (Supplemental Fig. 3). Of note, cancer-bearing animals that were treated with vehicle, but not QBKPN or QBECO, had, by Day 17 post-tumour inoculation, upregulated the expression of IL-10 – a cytokine associated with T regulatory cell function and cancer-induced immune suppression (Supplemental Fig. 3). In contrast, QBECO treatment was associated with increased levels of IL-7 and IL-15, which are cytokines related to T cell growth factor, IL-2, and are associated with both adaptive and innate immune lymphocyte function. This was surprising given QBECO did not reduce lung tumour burden (Fig. 1). We postulate that this lack of efficacy is at least in part due to the fact QBECO failed to induce significant chemokine production in the lungs resulting in fewer effector cells to control cancer growth. QBKPN’s anti-lung cancer efficacy was previously found to be dependent on the activation of the NKG2D pathway, which is fundamental for the detection and elimination of transformed, damaged and infected cells by cytotoxic lymphocytes^8^. QBKPN administration, but not QBECO administration, significantly potentiated the expression of NKG2D ligand, Rae1, in the lungs (Fig. 3A). In parallel, QBKPN treatment led to a more potent induction of molecules that mediate anti-tumour cytotoxicity including, Granzyme A (GzmA), Granzyme B (GzmB) and Perforin 1 (Pfr1), in the lungs of mice with cancer (Fig. 3B–E).

### The lung-specific immune response elicited by QBKPN is enhanced by the presence of cancer

**Figure 4:**
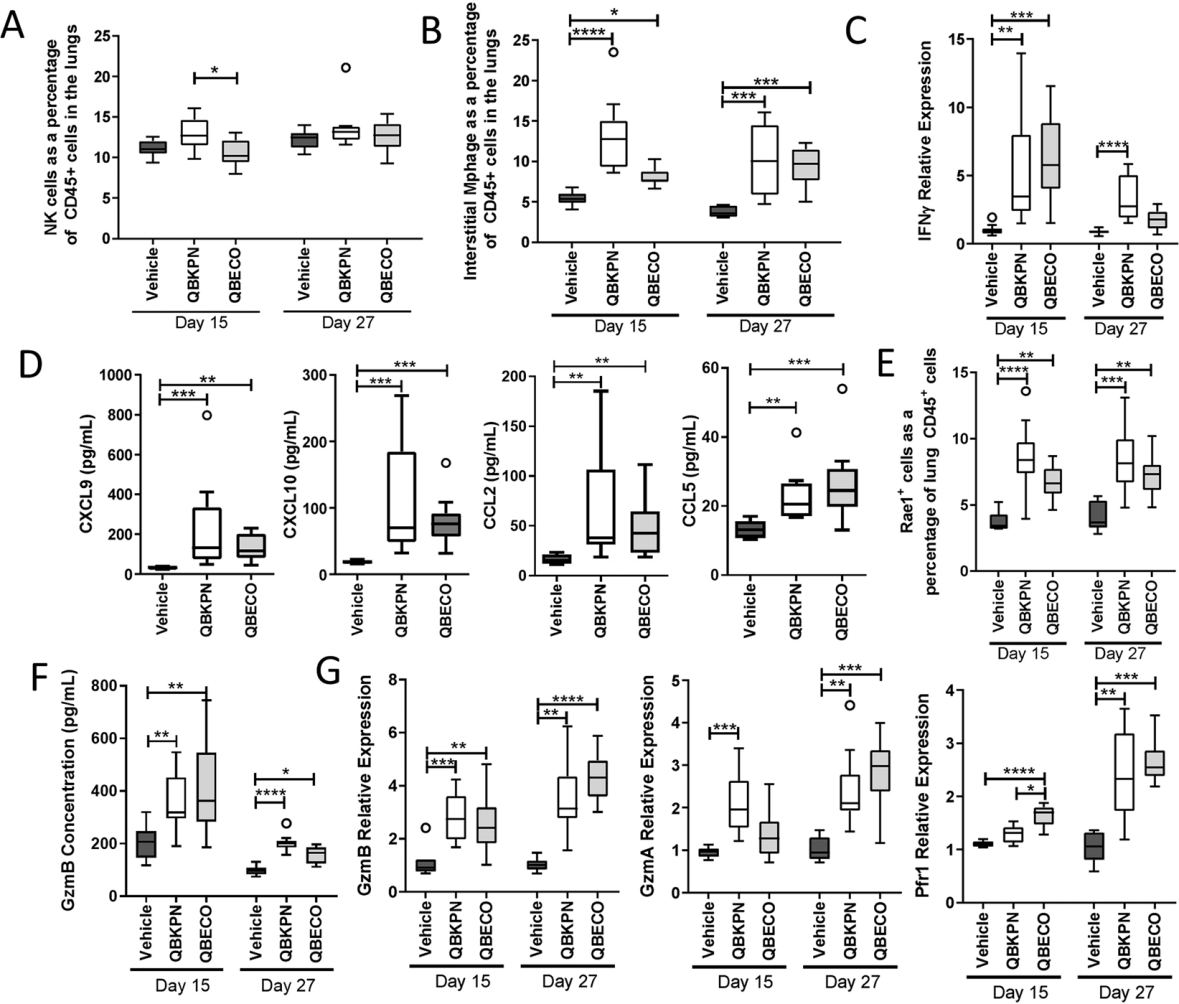
The lung enhanced response to QBKPN is limited in cancer free mice. (**A,B**) Lung immunophenotyping of cancer free C57Bl/6 mice after 15 and 27 days (which corresponds to the same treatment duration as day 5 and day 17 in the cancer models) of vehicle, QBKPN and QBECO treatment for **(A)** NK cells and **(B)** interstitial macrophages as a percentage of CD45^+^ cells. **(C)** IFNγ expression in the lungs tissue of cancer free mice. **(D)** Cytokine levels in lung homogenates for CXCL9, CXCL10, CCL2 and CCL5 in cancer free mice after 27 days of Vehicle, QBKPN and QBECO treatment. Cytokines were measured by 31-cytoplex; additional measured cytokines are in Supplemental Fig. 4. (**E)** Rae1^+^ expression on CD45^+^ cells in the lungs of cancer free mice, as assessed by flow cytometry. **F)** Protein levels of Granzyme B as measured by ELISA in the lungs of cancer free mice. **(G)** Gene expression in the lungs of cancer free mice for Granzyme B, Granzyme A and Perforin 1 measured by qRT-PCR. For all studies, data presented using Tukey boxplots. N = 9–10 mice per group. *p < 0.05; **p < 0.01; ***p < 0.001; ****p < 0.0001, Kruskal-Wallis test with Dunn’s multiple comparisons.

The contribution of the presence of cancer in the lungs to the type and extent of the immune response triggered by QBKPN vs. QBECO treatment was further investigated using cancer-free mice. Healthy animals had a slightly attenuated sustained recruitment of NK cells (assessed as a proportion of CD45+ cells) to the lungs with QBKPN treatment compared to lung cancer-bearing mice (Figs, 2A and 4A), particularly at the later time point. Whereas, the proportion of interstitial macrophages increased in both healthy mice and mice with cancer (Figs. 2B and 4B). There were differences between healthy and lung cancer-bearing mice in the degree to which certain immune responses were sustained. In particular, the expression of IFNγ, which decreased or remained the same over time in healthy mice (Fig. 4C), increased over time in cancer-bearing mice with QBKPN treatment (Fig. 2D). However, similar to cancer-bearing mice, there was generally greater lung immune stimulation with QBKPN than with QBECO treatment, but this difference tended to be less pronounced in healthy mice. The exception to this observation was in the gene expression, but not protein levels, of granzyme B, granzyme A, and perforin, which showed similar increases with 27 days of either QBKPN or QBECO treatment in cancer-free animals (Fig. 4G). Protein levels of granzyme B, however, were increased with QBKPN or QBECO at the latter 27 day timepoint only in mice with cancer **(**Fig. 3E**)**, but not in healthy mice **(**Fig. 4F**)**. Collectively, these findings suggest that the presence of disease likely influences the extent, duration and type of lung-specific immune response elicited by QBKPN vs. QBECO treatment.

### Both microbial-based treatments, QBKPN and QBECO, stimulate an acute immune response that results in emergency hematopoiesis

**Figure 5:**
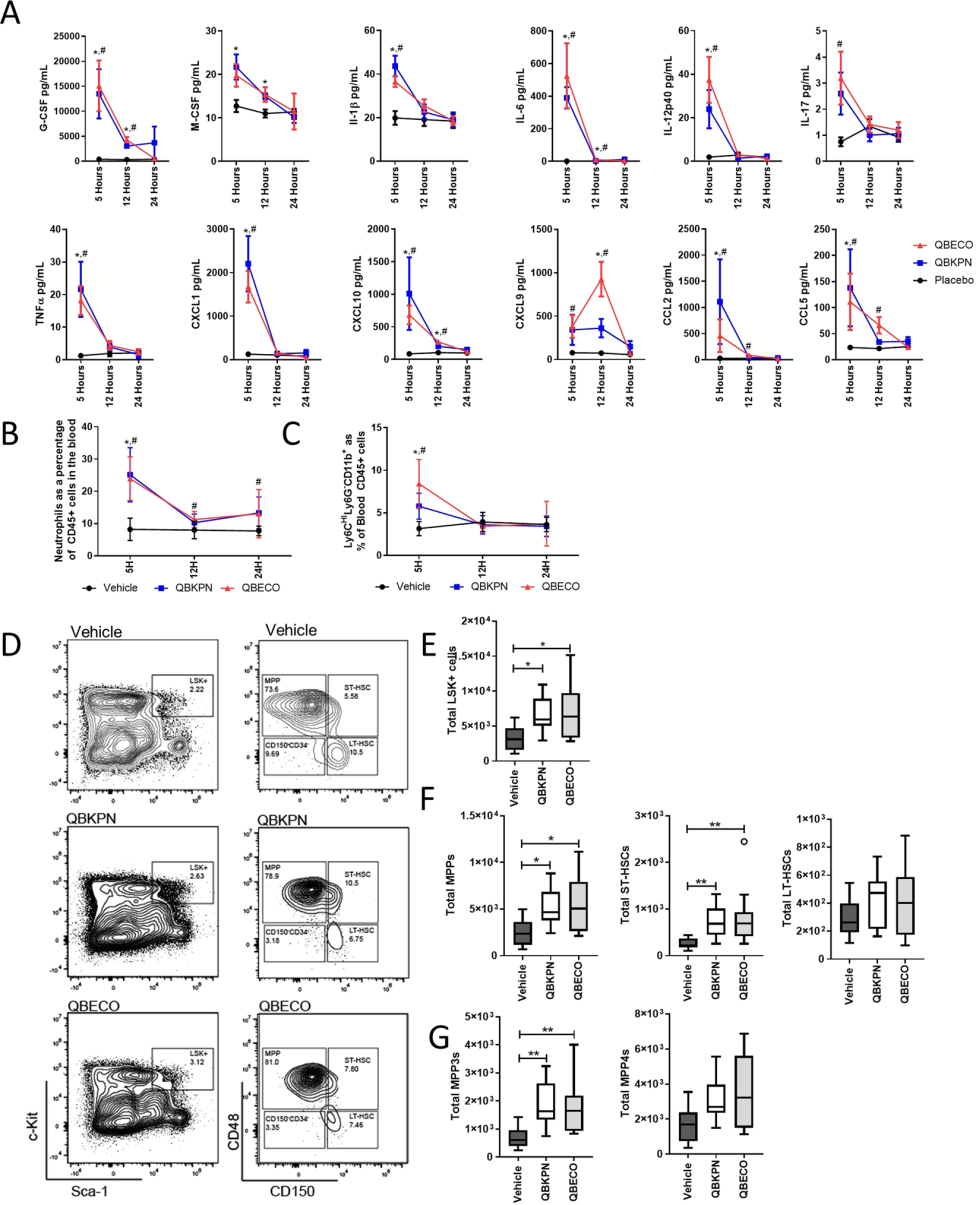
QBKPN and QBECO create an early cytokine response and stimulate emergency hematopoiesis. (**A**) Cytokine measurements in the serum 5, 12 and 24 hours after treatment with vehicle, QBKPN or QBECO by subcutaneous injection. Cytokines were measured by 31-cytoplex; additional measured cytokines are in Supplemental Fig. 5. N = 9–10 mice per group; mean ± SEM. Data from 5 hours in vehicle and QBKPN treated mice has been previously published^8^. **(B)** Neutrophils (Ly6G^+^Ly6C^−^) and **(C)** monocytes (Ly6C^HI^Ly6G^−^CD11b^+^) as a percentage of CD45^+^ blood cells. N = 10 mice per group. Mean ± SD shown. **(D)** Representative flow cytometry plots of the bone marrow 24 hours after mice were treated with a single dose of subcutaneous vehicle, QBKPN and QBECO for LSK+ (Lin^−^Sca-1^+^c-Kit^+^) and for multipotent progenitors (MPPs; CD150^−^CD48^+^), short-term HSCs (ST-HSC; CD150^+^CD48^+^), and long-term HSCs (LT-HSC; CD150^+^CD48^−^) cells. Cell counts of **(E)** LSK^+^ cells, **(F)** MPPs, ST-HSC, and HT-HSC, and **(G)** myeloid lineage MPPs (MPP3s; MPP^+^CD34^+^Flt3^−^) and lymphoid lineage MPPS (MPP4s; MPP^+^CD34^+^Flt3^+^) at 24 hours after a single subcutaneous dose of vehicle, QBKPN and QBECO. N = 10 mice per group. Mean ± SD shown. **(A–C)** *p < 0.05 for QBKPN vs vehicle; ^#^p < 0.05 for QBECO vs vehicle; ^%^p < 0.05 for QBKPN vs QBECO, Kruskal-Wallis test with Dunn’s multiple comparisons. **(E–G)** *p < 0.05; **p < 0.01, Kruskal-Wallis test with Dunn’s multiple comparisons.

The nature of the initial systemic immune response elicited by QBKPN and QBECO following a single subcutaneous injection was first assessed in healthy animals to exclude the impact of the presence of disease in the organ on their different organ-specific effects. The kinetics and magnitude of the systemic acute-immune response were similar after administration of the two microbial-based products (Fig. 5A–C). Both treatments induced a rapid induction of systemic inflammatory cytokines that were detectable in the serum within 5 hours and returned to baseline levels by 12 to 24 hours after administration (Fig. 5A; Supplemental Fig. 5). Similarly, both QBKPN and QBECO increased the number of neutrophils and Ly6C^HI^ monocytes in circulation within 5 hours. (Fig. 5B,C).

Changes in the hematopoietic stem cell (HSC) populations were investigated to assess how a single injection of either QBKPN or QBECO affected hematopoiesis and the mobilization of new immune cell recruitment. Twenty-four hours after treatment, both QBKPN and QBECO similarly expanded the HSC progenitor population in the bone marrow, characterized as lineage negative c-Kit^+^Sca^+^ (LSK^+^) (Fig. 5D,E). In particular, both microbial products increased the production of short-term HSC (ST-HSC; characterized as LSK^+^CD150^+^CD48^+^) and multipotent progenitors (MPPs; LSK^+^CD150^−^CD48^+^); Fig. 5G. Myeloid (MPP3; LSK^+^CD150^−^CD48^+^CD34^+^Flt3^−^) lineages were significantly enhanced at this time point with QBKPN and QBECO treatment, as were lymphoid (MPP4; LSK^+^CD150^−^CD48^+^CD34^+^Flt3^+^) lineages, but to a lesser extent.

### In lung cancer bearing mice, QBKPN-mediated myelopoiesis induces recruitment of trained innate immune cells to clear pathology

**Figure 6:**
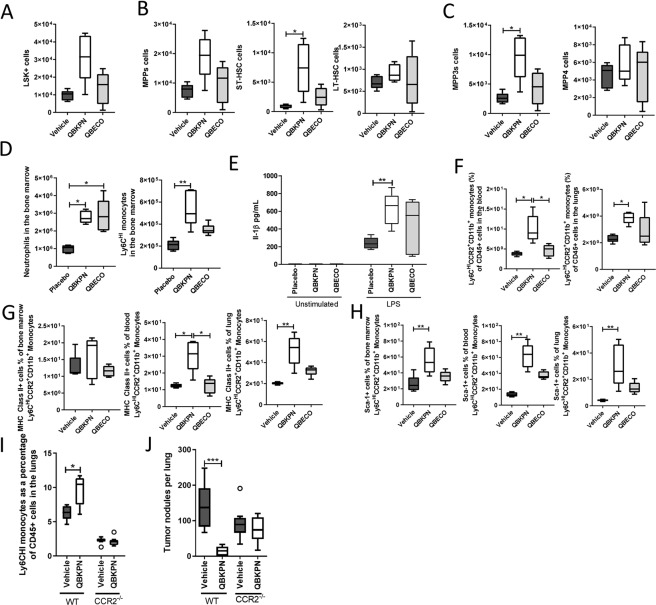
Repeated administration of QBKPN in the context of lung cancer promotes myelopoiesis and induction of trained monocytes. (**A–C**) Cell counts of LSK^+^ cells, MPPs, ST-HSC, HT-HSC, CD150^+^CD45^−^LSK^+^ cells, MPP3s (MPP^+^CD34^+^Flt3^−^) and MPP4s (MPP^+^CD34^+^Flt3^+^) in B16F10 bearing C57BL/6 mice 18 days after tumour inoculation, and administered vehicle, QBKPN or QBECO. N = 5 mice per group. **(D)** Total number of neutrophils and Ly6C^HI^ monocytes in the bone marrow in the day 18 B16F10 lung cancer model. N = 5 mice per group. **(E)** IL-1β levels in the supernatant of bone marrow monocytes originally isolated from mice in the day 17 B16F10 lung cancer model and subsequently stimulated for 24 hours with LPS (1 ng/mL) or left unstimulated. N = 5 mice per group; analysis was between microbial-based treatment (QBKPN or QBECO) and vehicle. No IL-1β production was detected in the unstimulated samples. **(F)** Ly6C^HI^CCR2^+^CD11b^+^ monocytes as a percentage of all CD45^+^ cells in the blood and lungs at day 18 in the B16F10 cancer model. N = 5 mice per group. **(G)** MHC Class II expression on Ly6C^HI^CCR2^+^CD11b^+^ monocytes in the bone marrow, blood and lungs at day 18 in the B16F10 cancer model. N = 5 mice per group. **(H)** Sca-1 expression on Ly6C^HI^CCR2^+^CD11b^+^ monocytes in the bone marrow, blood, and lungs at day 18 in the B16F10 cancer model. N = 5 mice per group. **(I)** Monocytes (Ly6C^HI^CD11b^+^Ly6G^−^) and **(J)** tumour burden, in the lungs at day 15 in B16F10 cancer bearing wildtype (WT) and CCR2 knockout (CCR2^−/−^) mice administered with QBKPN or vehicle. N = 8 mice per group. In each experiment, mice were treated with vehicle, QBKPN, or QBECO by subcutaneous injections every second day starting 10 days before tumour seeding and continuing throughout the experiment. Data presented as Tukey boxplots. *p < 0.05; **p < 0.01; ***p < 0.001, Kruskal-Wallis test with Dunn’s multiple comparisons **(A–C**,**F–H)**, Mann-Whitney test **(E**,**I–J)**.

Microbial-based stimulation of myelopoiesis is considered to be an important component of trained innate immunity required to deal with the threat of infection^13^. To determine whether this process is also involved in mediating QBKPN’s ability to clear cancer in the lungs, the modulation of myelopoiesis by QBKPN and QBECO was investigated in the context of cancer in the lungs. Mice repeatedly treated with QBKPN, but not mice treated with QBECO, had sustained increases in the number of HSC (LSK^+^ cells) compared to vehicle-treated control mice when the bone marrow niche was assessed 18 days after B16F10 cancer cell administration seeded to the lungs (Fig. 6A–C). This QBKPN-mediated increase in progenitors favoured MPPs and ST-HSCs (Fig. 6B,C). Within the MPP population, repeated treatment with QBKPN in lung cancer-bearing mice resulted in a shift towards MPP3 generation (Fig. 6C). In contrast, QBECO treatment had little effect in modulating the HSC populations in lung-cancer bearing mice when assessed at this later time point (Fig. 6A–C). This difference in the ability to sustain hematopoiesis between the two microbial-based treatments at the later time point (Day 18 post-tumour) is, we believe, attributable to the fact that unlike QBKPN, QBECO treatment failed to reduce cancer-burden in the lungs (Fig. 1A). As a result, the progression of lung cancer and deteriorating health and immune status in mice treated with QBECO results in an inability of QBECO treatment to sustain the induction of myelopoiesis to the same extent as QBKPN in mice with lung cancer. Both QBKPN and QBECO treatment induced large increases in bone marrow neutrophil numbers; however, only QBKPN was able to sustain an increased number of bone marrow monocyte numbers (Fig. 6D).

Finally, to test whether bone marrow monocytes in mice treated with these microbial-based therapies were functionally different (i.e. trained), we isolated bone marrow monocytes from B16 lung tumour bearing mice treated with vehicle, QBKPN or QBECO and stimulated them with LPS *in vitro*. Bone marrow monocytes from mice treated with QBKPN or QBECO released more IL-1β relative to those treated with vehicle. (Fig. 6E), as would be expected from trained monocytes^14^.

In corollary to the findings on hematopoietic cells in the bone morrow, when we investigated systemic immune cell populations in the lung cancer model at Day 18, we found QBKPN, but not QBECO, treatment was able to sustain the increased numbers of Ly6C^HI^CCR2^+^CD11b^+^ monocytes in circulation and lungs (Fig. 6F). These monocytes were characterized as being positive for MHC class II and Sca-1 (Fig. 6G–H), markers associated with transcriptional programming in the bone marrow by microbial stimulation^15^. Ly6C^HI^ monocytes are recruited to sites of infections through CCR2/CCL2 signalling^16,17^. The important contribution to anti-lung cancer efficacy of QBKPN-mediated recruitment of these monocytes was demonstrated in mice genetically deficient in CCR2, in which QBKPN treatment failed to recruit Ly6C^HI^ monocytes to the lungs and lost its anti-cancer efficacy (Fig. 6I,J).

### Demonstration of the broader application of immune modulation by organ-specific microbial-based therapies to treat immune-related diseases

The ability to target trained innate immune cells to specific organ sites of pathology by the strategic selection of the correctly matched microbial-based therapy has potential to treat a broad range of immune-related diseases. Adding to the evidence provided for this novel approach for cancer using specific gram-negative pathogens targeting the lungs, peritoneal cavity and GI tract, we next tested the ability to target the skin using a microbial-based treatment (QBSAU) made from a clinical isolate of *Staphylococcus aureus*, a Gram-positive organism that is a common source of skin infection. Treatment with QBSAU, but not QBKPN or QBECO, was effective in reducing B16F10 melanoma subcutaneous tumour growth (Supplemental Fig. 6A). In contrast, QBSAU had little relative efficacy in improving outcomes for animals with cancer in the lungs or peritoneal compartment (Supplemental Fig. 6B,C).

**Figure 7:**
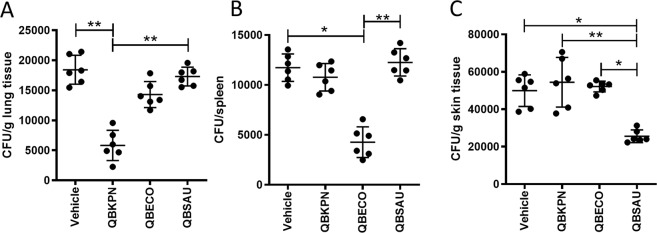
Microbial-based treatment protect against infections in an organ-specific manner. (**A**) Lung bacterial counts 3 days after mice were challenged with *Streptococcus pneumoniae* by intratracheal instillation and treated with vehicle, QBKPN, QBECO or QBSAU. **(B)** Spleen bacterial counts in mice challenged with *Salmonella enterica (typhimurium)* by intraperitoneal injection and treated with vehicle, QBKPN, QBECO or QBSAU. **(C)** Skin bacterial counts 3 days after mice were challenged with a subcutaneous infection of *Pseudomonas aeruginosa* and treated with vehicle, QBKPN, QBECO or QBSAU. In each experiment, treatments were given by subcutaneous injections every second day as described in the Material and Methods. N = 6 mice per group. Data is shown with mean ± SD. *p < 0.05; ***p < 0.001; ****p < 0.0001, Kruskal-Wallis test with Dunn’s multiple comparisons.

To demonstrate the broader application of this immunotherapeutic approach, we tested the three different microbial-based treatments, QBKPN, QBECO, and QBSAU, in three infection models affecting the lungs, peritoneal cavity and skin. Pathogens different from those used to make the microbial-based treatments were used for establishing organ-specific infections to avoid the possibility that the treatments were functioning like classical vaccines through the induction of an adaptive immune response to a specific pathogen. In the *Streptococcus pneumoniae* lung infection model, out of the three microbial-based treatments used, QBKPN performed the best in reducing the bacterial burden in the lungs (Fig. 7A). In corollary, the intraperitoneal *Salmonella enterica* infection model showed that QBECO treatment had the greatest efficacy in reducing the peritoneal bacterial burden as assessed in the spleen (Fig. 7B). Lastly, in the *Pseudomonas aeruginosa* skin infection model, QBSAU treatment was the most effective in reducing the skin bacterial burden (Fig. 7C). The ability of these microbial-based therapies to ameliorate disease was independent of the species of pathogen causing infection. QBKPN reduced bacterial burden in the context of both *S. pneumoniae* and *P. aeruginosa* (Supplemental Fig. 7) infections.

## Discussion

Microbial exposure is a double-edged agent of health and disease. Infection was once the leading cause of morbidity and mortality, but with significant improvements in hygiene and the advent of population-based vaccination and antibiotics, the 20^th^ Century saw a dramatic decrease in the incidence of acute infection and infection mortality and a concomitant rise in allergies, chronic immune-related disorders^18,19^ and certain cancers^20^ in developed countries. This observation has become popularly known as the hygiene hypothesis, which postulates that a lack of appropriate training of the immune system by microbial stimulation, particularly early in life, is linked to the development of immune pathologies. This hypothesis has different variations, some postulating that exposure to disease causing agents is less important than early life exposure to “friendly” or symbiotic microbes^21^. The truth likely encompasses an integration of both concepts. Evidence for the hygiene hypothesis has primarily come from epidemiological studies (reviewed in^18,19,22,23^), but there is direct experimental evidence that immune education by microbial stimulation early in life is important for setting the tone for immune regulation throughout^24^. This has spurred an area of avid research focused on the use of microbes and microbial components as potential new treatment modalities for the many serious immune-related diseases growing in incidence and prevalence^5,25,26^. In this work, we show the potential of using different pathogen-based stimuli to harness the immunological memory contained within specific regional niches to overcome pathology by formulating the pathogen-based treatment from a microbe that is a likely endogenous source of infection of the target tissue.

Collectively, the studies presented suggest that the choice of microbe used to formulate an immune modulator may play an important role in appropriately directing innate immune effector cells to the site of disease. We hypothesize this differential immune targeting by different microbial-based subcutaneously administered stimuli is a form of resource management used by the immune system upon the first threat of an acute infection. It is important that this stimulus is perceived as an acute threat by the immune system to rapidly mobilize the effector functions we describe. We know from our developmental studies that this mobilization can be achieved by subcutaneous injection, but not when the microbial stimulus is delivered orally or by inhalation. After injection of the microbial stimulus, it appears immune cells are activated to rapidly survey the body for the potential source of the pathogen administered to send a newly primed army to the site where that kind of microbe is found in greatest abundance and is the likely source of infection. Each of the microbial-based stimulants we used are organisms that are endogenous in certain organ sites, and we know from previous work that prior exposure to that live microbe (by either overt or subclinical infection) is necessary for efficacy^8^. We are thus able to successfully target disease in the lungs, peritoneal cavity, GI tract and skin by using distinct microbial stimulants presented as an acute threat that are associated with each of those respective sites.

**Figure 8:**
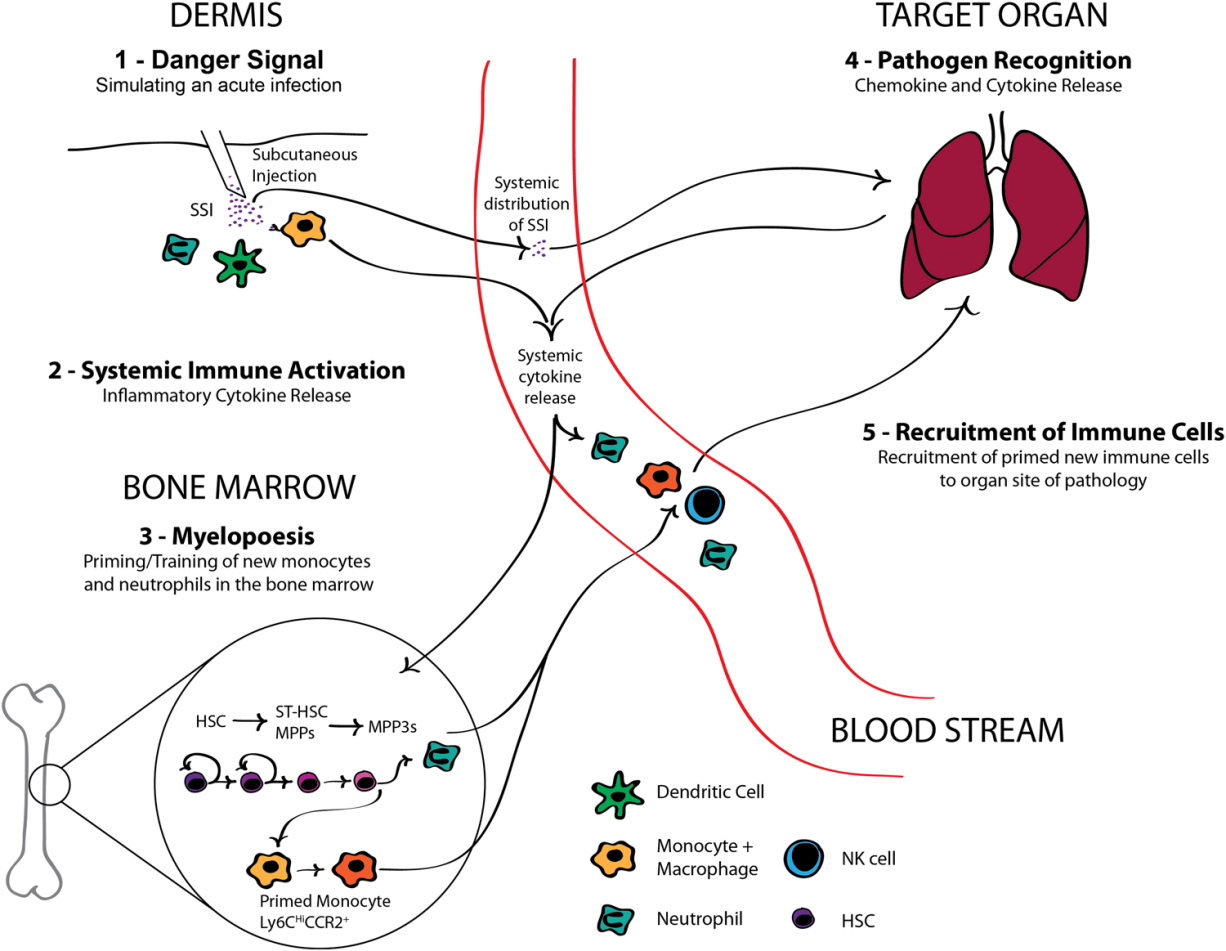
Summary of the mechanism leading to organ-specific immune activation by a subcutaneously delivered microbial stimulus. The detection of microbial components delivered subcutaneously is perceived as an acute threat. In response, the immune system is mobilized to deal with this threat by triggering emergency myelopoiesis, a process that results in training (or programming) new immune cells in the bone marrow to deal effectively with the presence of any malignancy or breach in defences. In parallel, the microbial components that reach the target organ bearing pathology are presented in the context of an immunological threat. In the illustration, the target organ is represented by the lungs, but it can be any of the tissue sites we targeted including the peritoneal cavity and skin. When the microbial components match pathogens that are common in the target organ, local production of chemokines is induced that recruit the newly trained immune cells coming out of the bone marrow. The end result is a targeted and efficient immune response to the pathology present in the targeted organ.

The lack of consistency and high variability in outcomes with microbial-based treatments to date may partly be due to a mismatch between the microbial species from which the treatment is derived and the past microbial exposure or “training” of the patient’s immune system in the targeted organ. Figure 8 summarizes the sequence of events we believe leads to a site-specific amelioration of pathology using a microbial-based treatment derived from a pathogen that is a common cause of infection in that organ niche.

Two long-held dogmas have recently been brought into question with paradigm-shifting advances in our understanding of innate immunity: (1) immunological memory is the domain of the adaptive arm of the immune system, and (2) innate immunity lacks specific memory^5,6,27,28^. The treatment strategy we employed is rooted in both the trainability of the innate immune system and its more nuanced organ specificity, which is intrinsically different from that of the adaptive immune system^27^. We found that subcutaneous administration of a microbe-based stimulant results in myelopoiesis-associated training of monocytes in the bone marrow within 24 hours. Intraperitoneal administration of a prototypical agonist for innate immune training, β-glucan, was first shown to act on HSC to expand myeloid cell progenitors, and this effect on HSC improved the physiological response to subsequent challenge with LPS and also protected mice from chemotherapy-induced myelosuppression^13^. This work by Mitroulis and colleagues established that microbial modulation of myelopoiesis is a fundamental component of trained immunity^13^. Kaufmann and colleagues had come to a similar conclusion in parallel using BCG in a series of elegant experiments that demonstrated BCG administration induced myelopoiesis at the expense of lymphopoiesis and the production of epigenetically modified macrophages that were better able to deal with virulent M. tuberculosis^29^. However, their intent was to demonstrate the longer-term benefits of innate immune conditioning, enabling it to deal more effectively with challenges it will face in the future. We have shown that this innate immune training in the bone marrow can be therapeutically harnessed to deal with a potentially broad spectrum of imminent or present immunological threats by targeting the recruitment of these cells to a specific organ.

Askenase and colleagues discovered that the priming of monocytes in the bone marrow in response to infection was mediated by the production of IFNγ by NK cells, and this proactive developmental education promoted their effector functions in the infected tissues^15^. Microbial-induced innate immune conditioning in the bone marrow is governed by secreted inflammatory signals, including IL-1β, GM-CSF, IL-12 and IFNγ, and largely lacks specificity^13,15^. As we demonstrated, primed monocytes, once recruited to the tissues, can potently respond to a range of dangers. This is consistent with studies demonstrating that BCG vaccination offers non-specific protection against non-mycobacterial infections that is independent of adaptive immune function^30^. In our study, we observed that there appears to be a chemokine-mediated gradient for targeting immune cells newly released from the bone marrow to tend to go to the organ where direct interaction with the microbial species occurs. Given the wide sampling of microbial organisms in the body, this may be a simple matter of directing the focus where the organism is most abundant, as hypothesized above. Alternatively, it may be that different immunological niches are trained to respond to specific pathogens differently based on previous exposure. Olszak *et al*. demonstrated that early life microbial exposure modulated the recruitment of invariant NKT cells to mucosal sites later in life^24^, however, the mechanism by which this microbial regulation is instituted was not defined.

It makes intuitive sense that there exists a means by which the innate immune system directs its resources to the most likely source of infection upon detecting microbial components that are presented as an acute threat. We propose this organ-specific microbial recognition reflects a more sophisticated level of immunological education than the non-specific training that initially occurs in the bone marrow. It is reminiscent of a similar phenomenon observed in invertebrates, which lack adaptive immunity, yet show a stronger immune response to bacterial strains previously encountered compared to strains to which they are naïve^27^. It was previously demonstrated that a *Klebsiella*-based immunotherapy was effective in reducing lung cancer burden in Rag2 deficient mice that are largely devoid of classical adaptive immune function^8^. The contribution of adaptive immunity to the observed microbial mediated organ-specificity cannot be ruled out; however, we believe it is unlikely the primary driver of this organ specific memory and recruitment. Cells not conventionally considered to be part of the immune system, such as epithelial and endothelial cells that function as the interface between the host and its microbiota, are also capable of forming long-term memories. Acute inflammation was elegantly shown to functionally change epithelial stem cells to respond to such subsequent assaults with improved barrier restoration^31^. On the other hand, barrier dysfunction can also develop through reprogramming of epithelial cells in response to allergic inflammation^32^. This epithelial inflammatory memory, similar to innate immune training^5^, is mediated at the level of epigenetic programming^31^. In our study, the presence of pathology in the targeted tissue further potentiated this organ specific immune response to the microbial stimulus, and the nature of the pathology dictated the effector functions executed by the recruited primed innate immune cells.

The therapeutic potential of trained innate immunity has yet to be fully realized. Recent advances in understanding the basis of innate immune programming has re-ignited research into the application of microbial modulation of immune function for the prevention and treatment of disease. The discovery of tissue-specific microbial memory that can be therapeutically exploited to direct trained innate immune effector cells to sites of pathology has the potential to change the way diseases rooted in immune dysfunction are treated. In addition to treating various cancers and infections, proof-of-principle experiments conducted to date show promise for this approach for the treatment of asthma^33^, COPD^34^, and inflammatory bowel disease^35^. The clinical application of this immunotherapeutic strategy has been promising thus far, based on Phase 1 and 2 studies in non-small cell lung cancer^8^, ulcerative colitis^36^, and Crohn’s disease^37^. Further research is now needed to fully elucidate the detailed immunological mechanisms of the organ specific innate immune response to microbial threat and its therapeutic application.

## Materials and Methods

### Animals

Female C57BL/6J mice (aged 6–10 weeks) were sourced from Jackson Laboratories (Bar Harbor, ME, USA) and Envigo (Livermore, CA, USA). Female CCR2-deficient mice (B6.129S4-Ccr2 < tm1lfc > /J) were purchased from Jackson Laboratories. Mice were acclimatized and housed for at least one additional week prior to the experimental studies and were contained in environmentally controlled conditions with a 12:12 hour light/dark cycle for the duration of the study.

### Ethics statement

All animal care procedures and experimental protocols were approved by the Institutional Animal Care and Use Committee (IACUC) of Dartmouth College (protocol 1141). All animal care procedures and experimental protocols were performed in accordance with applicable international, national, and/or institutional guidelines, including NIH and USDA policies on the care and use of animals in research and teaching. All experimental protocols involving animals performed at Qu Biologics were reviewed and approved by their Animal Care Committee under AUP-201001-001 and were conducted in adherence to the Canadian Council on Animal Care’s (CCAC) policies and guidelines.

### Microbial-based treatments

All the microbial-based therapies used in this study were made from clinical isolates that are the property of Qu Biologics Inc (Vancouver, Canada). QBKPN is a proprietary investigational immunotherapeutic formulated from an inactivated clinical lung isolate of *Klebsiella variicola* acquired from a patient with pneumonia^8,33,34^. QBECO is a similarly manufactured proprietary investigational immunotherapeutic made from an inactivated clinical isolate of *Escherichia coli* from a patient with bloody diarrhea. QBSAU is a proprietary investigational immunotherapeutic made from an inactivated clinical isolate of *Staphylococcus aureus* acquired from a patient with an acute subcutaneous abscess. QBKPN, QBECO and QBSAU are suspended in physiological saline, with or without 0.45% phenol as a preservative, and were supplied by Qu Biologics. The vehicle control used was physiological saline, with or without 0.45% phenol. Requests for any of the materials used for treatment in this study should be directed to the corresponding author, HG. For experiments performed using only a single dose, 30 μL of the treatment solution was subcutaneously injected into the lower right abdomen. For longer-term experiments involving multiple treatments, subcutaneous injections (into the animal’s skin folds) were administered every second day, rotating among the lower right abdomen, upper right chest, upper left chest, and lower right abdomen of the animal. For cancer model experiments in which the cancer cells were injected into the animal to seed, treatment starting 10 days before tumour cell inoculation (day 0) and every second day throughout the experiment except for the day of tumour inoculation, as previously described^8^. For experiments performed using the DSS/AOM cancer model and naïve mice, the microbial-based treatments were given every second day throughout the experiment. For the infection models, treatment started 14 days before bacterial challenge (day 0) and was given every second day throughout the experiment, except for the day of bacterial challenge.

### Cancer cell injection models

B16-F10 melanoma cells (ATCC CRL-6475, Manassas, VA, USA), red fluorescent protein (RFP)-tagged Lewis lung carcinoma (LLC-RFP) cells (AntiCancer Inc.), MC-38 adenocarcinoma cells (gift from Dr. Jeff Schlom, National Cancer Institute), and Panc02 pancreatic cancer cells (gift from Professor Dieter Kabelitz, Institute of Immunology, Kiel University) were cultured in RPMI medium supplemented with 10% FBS. Cancer cells were harvested and resuspended in PBS before implantation. Cells were confirmed to be free of Mycoplasma infection using the Hek Blue Mycoplasma Detection Kit from Invivogen.

For the lung cancer studies, cells suspended in 100 ul of PBS were injected intravenously by tail vein injection using 2 × 10^5^ B16-F10 cells and 4 × 10^5^ LLC-RFP cells. Tumour burden was assessed at day 15 post-tumour inoculation by enumerating the visible surface metastatic lung lesions; LLC-infiltrated lungs were stained with Bouin’s solution (Sigma-Aldrich, St. Louis, MO, USA) to provide contrast.

For the intraperitoneal cancer survival studies, MC-38 cells or Panc02 cells were injected intraperitoneally at 2 × 10^5^ cells in 100 µL PBS. Mice were monitored daily and euthanized when they reached humane endpoint criteria (i.e., appearance, posture/gait, abdominal distension, behavioural and facial signs of pain).

For the skin cancer study, B16-F10 cells were injected subcutaneously at 1 × 10^5^ cells in 100 µL PBS. Tumour length, width, and height were measured by digital calipers and the tumour burden was calculated using these measurements every second day.

### DSS/AOM model

Mice were injected intraperitoneally on day 0 with azoxymethane (AOM; Sigma, Kawasaki, Kanagawa Prefecture, Japan), at 8 mg/kg body weight, 10 days after treatment started. After AOM injections, mice received 3 cycles of dextran sodium sulphate (DSS) (MP Biomedicals, Santa Ana, CA, USA) treatment spaced 3 weeks apart. In each cycle, DSS was administered in the drinking water *ad libitum* for 5 days at a final concentration of 2.5% weight/volume. Mice were euthanized at the experimental endpoint 70 days after AOM injection.

### Biodistribution imaging of fluorescently labelled QBKPN

The National Research Council (NRC) of Canada performed the *in vivo* biodistribution studies. Labelling of the microbial-based product, QBKPN, was carried out using Cy5.5 NHSester (GE Healthcare Life Sciences). All optical imaging experiments were performed using a small-animal time-domain eXplore Optix MX2 pre-clinical imager, and images were analyzed or reconstructed as fluorescence concentration maps using ART Optix Optiview analysis software 2.0 (Advanced Research Technologies, Montreal, QC). The pharmokinetic studies were conducted by collecting blood samples, approximately 0.08 mL, from the submandibular vein at selected time points (from 10 minutes to 96 hours after administration) using three mice.

### Flow cytometry

Anti-mouse antibodies CD16/32, CD45 (30-F11), CD4 (GK1.5), CD8 (53–6.7), NK1.1 (PK136), Ly6G (1A8), Ly6C (HK1.4), F4/80 (BM8), CD11b (M1/70), MHC class II (M5/114.15.2), Rae1 (CX1), CD64 (X54-5/7.1), CD24 (M1/69), CD11c (N418), CCR2 (SA203G11), Sca1 (E13-161.7), CD150 (TC15-12F12.2), CD48 (HM48-1), CD34 (RAM34), Flt3 (A2F10) and c-kit (2B8) were sourced from BioLegend (San Diego, CA, USA) and eBioscience (Waltham, Massachusetts, USA). For HSC determination, lineage cells were stained by Mouse Lineage Antibody Cocktail (Catalog Number 561301, BD Biosceince). Lungs were harvested, specific lobes were removed for protein and gene expression, and the remainder was dissociated into a single cell suspension using the Milltenyi Biotec (San Diego, CA, USA) mouse lung dissociation kit and gentleMacs tissue dissociator. Bone marrow cells were harvested from both femurs and tibias. Red blood cells were removed from blood, lung and bone marrow samples using Red Blood Cell Lysis Buffer (Biolegend) before staining. Cell viability was assessed using the live/dead fixable violet cell stain kit (ThermoFisher Scientific) and for HSC sample analysis, CD16/32 was blocked using TruStain fcX (BIoLegend). Flow cytometry was performed on a CytoFlex (Bechman Coulter, Indianapolis, IN, USA). Data were analyzed using FlowJo software (version 10.2, Ashland, OR, USA). Lung immune cell profiles were created using a protocol modified from Yu *et al*.^38^ while bone marrow hematopoietic stem cells populations were gated as defined by Kaufmann *et al*.^29^.

### Immune mediator profiling

Right lung caudal lobes were homogenized using TissueLyser LT (Qiagen, Hilden, Germany) in 0.5 mL of 1% Tween-20 (Sigma-Aldrich, St. Louis, MO, USA) solution. Serum and lung immune factors were analyzed by multiplex technology (performed by Eve Technologies, Calgary, AB, Canada) using a 31 cytokine/chemokine/growth factor kit (Millipore, St Charles, MO, USA). The assay was run on a Bio-Plex™ 200 system (Bio-Rad Laboratories, Inc., Hercules, CA, USA). Data from 5 hours in vehicle and QBKPN treated mice has been previously published^8^.

### Gene expression

The right lung post-caval lobe was homogenized by a TissueLyser LT (Qiagen, Toronto, ON, Canada). RNA was isolated using the PureLink RNA Mini Kit (Life Technologies, Carlsbad, CA, USA) and reverse transcribed into cDNA using the iScript cDNA Synthesis Kit (Bio-Rad). Gene expression was quantified by quantitative RT-PCR using a StepOnePlus RT-PCR machine (Applied Biosystems, Foster City, CA, USA), TaqMan Fast Advanced Master Mix (Applied Biosystems) and Taqman probes (Applied Biosystems) for granzyme A (Mm01304452_m1), granzyme B (Mm00442837_m1), perforin (Mm00812512_m1) and IFNγ (Mm01168134_m1). All genes were normalized against GAPDH (Mm99999915_g1).

### Granzyme B protein analysis in lung samples

Right lung caudal lobes were homogenized by TissueLyser LT and processed as per the manufacturer’s protocol (Qiagen). Granzyme B protein was quantified by ELISA according to the manufacturer’s protocol (eBioscience).

### *In vitro* stimulation of bone marrow monocytes

Bone marrow cells were harvested from both femurs and tibias. Monocytes were isolated using an EasySep^TM^ Mouse Monocytes Isolation Kit (STEMCELL, Vancouver, BC, Canada). 30000 monocytes plated per well in a round bottom 96 well plate in RPMI 1640 with 10% FPS and penicillin-streptomycin. Cells were either unstimulated or challenged with LPS (1ug/mL; LPS from *E. coli* O111:B4; Sigma-Aldrich) for 24 hours. Supernatant was collected and IL-1β levels were measured by ELISA (R&D systems, Minneapolis, MN, USA).

### Infection models

*Pseudomonas aeruginosa* (PA14), *Streptococcus pneumoniae* (P1121) and *Salmonella enterica (typhimurium)* (CDC 6516-60) were individually grown aerobically overnight in Luria Broth (LB), pelleted, and resuspended in PBS to the appropriate concentration based on optical density (OD_600_). Mice were treated with one of QBKPN, QBECO, QBSAU or vehicle starting 14 days before bacterial challenge and administrations were given every second day throughout the experiment, except for the day of bacterial challenge. On day zero, mice were challenged with the appropriate bacteria. For the lung infection models, mice were challenged with *S. pneumoniae* or *P. aeruginosa* by intranasal instillation of 5.0 × 10^5^ CFU^39^. For the intraperitoneal injection models, mice were challenged with 1 × 10^6^ CFU of *S. enterica (typhimurium)* bacteria by IP injection^40,41^. For the skin infection model, mice were challenged with *P*. *aeruginosa* by intradermal injection of 6.5 × 10^6^ CFU of bacteria^42^. On day three post-challenge, mice were sacrificed and the targeted organ (skin, lungs, or spleen) was aseptically resected, weighed, homogenized, and assessed for bacterial load. Counts were performed on LB agar plates for *S. pneumoniae*, *Hektoen* enteric agar plates^43^ (Becton Dickinson, Franklin Lakes, NJ, USA) for *S. enterica (typhimurium)* and *Pseudomonas* selection agar plates (Teknova, Hollister, CA, USA) for *P. aeruginosa*.

### Data analysis

GraphPad Prism 6 Software (GraphPad Software, San Diego, CA, USA) was used to perform statistical analyses. Data graphed as dot plots (Figs. 1 and 7) and serum cytokines in Fig. 5 are presented with the mean ± SD. However, to avoid making assumptions about distributions and given the high variability in many of the measures, we used non-parametric tests (Kruskal-Wallis or Mann Whitney, as indicated) for the majority of the statistical analyses; the only exception is for assessing the change in tumour volume over time (Supplemental Fig. 6) for which we used a repeated measures test. Boxplots in Figs. 2–6 are Tukey boxplots. Post-hoc analysis following use of the Kruskal-Wallis Test uses Dunn’s Multiple Comparisons Test. Differences in survival time was determined by Log-rank (Mantel-Cox) analysis. Outliers in tissue cytokines were identified by GraphPad’s ROUT test. Statistical significance was set at α level of 0.05.

## Supplementary information

Supplementary Data.

## References

[1] Power D (1899). The local distribution of cancer and cancer houses. Practitioner.

[2] Jessy T (2011). Immunity over inability: The spontaneous regression of cancer. J. Nat. Sci. Biol. Med..

[3] Thomas JA, Badini M (2011). The role of innate immunity in spontaneous regression of cancer. Indian J. Cancer.

[4] Brausi M, Olaru V (2012). Management of high-risk non-muscle invasive bladder cancer. Minerva Urol. Nefrol..

[5] Netea MG (2016). Trained immunity: A program of innate immune memory in health and disease. Science.

[6] Netea MG (2013). Training innate immunity: the changing concept of immunological memory in innate host defence. Eur. J. Clin. Invest..

[7] Netea MG, Joosten LAB, van der Meer JWM (2017). Hypothesis: stimulation of trained immunity as adjunctive immunotherapy in cancer. J. Leukoc. Biol..

[8] Bazett, M. *et al*. Harnessing innate lung anti-cancer effector functions with a novel bacterial-derived immunotherapy. *Oncoimmunology* **7**, 10.1080/2162402X.2017.1398875 (2017). 10.1080/2162402X.2017.1398875 PMC5790356 29399400

[9] Feng X (2014). Escherichia coli Peritonitis in peritoneal dialysis: the prevalence, antibiotic resistance and clinical outcomes in a South China dialysis center. Perit. Dial. Int..

[10] Obermajer N (2018). Promoting the accumulation of tumor-specific T cells in tumor tissues by dendritic cell vaccines and chemokine-modulating agents. Nat. Protoc..

[11] Thaker, A. I., Shaker, A., Rao, M. S. & Ciorba, M. A. Modeling colitis-associated cancer with azoxymethane (AOM) and dextran sulfate sodium (DSS). *J. Vis. Exp*. **67**, 10.3791/4100 (2012). 10.3791/4100 PMC3490277 22990604

[12] Parang B, Barrett CW, Williams CS (2016). AOM/DSS Model of Colitis-Associated Cancer. Methods Mol. Biol..

[13] Mitroulis I (2018). Modulation of Myelopoiesis Progenitors Is an Integral Component of Trained Immunity. Cell.

[14] Arts RJW (2018). BCG Vaccination Protects against Experimental Viral Infection in Humans through the Induction of Cytokines Associated with Trained Immunity. Cell. Host Microbe.

[15] Askenase MH (2015). Bone-Marrow-Resident NK Cells Prime Monocytes for Regulatory Function during Infection. Immunity.

[16] Kurihara T, Warr G, Loy J, Bravo R (1997). Defects in macrophage recruitment and host defense in mice lacking the CCR2 chemokine receptor. J. Exp. Med..

[17] Shi C, Pamer EG (2011). Monocyte recruitment during infection and inflammation. Nat. Rev. Immunol..

[18] Bach JF (2002). The effect of infections on susceptibility to autoimmune and allergic diseases. N. Engl. J. Med..

[19] von Mutius E (2007). Allergies, infections and the hygiene hypothesis–the epidemiological evidence. Immunobiology.

[20] Rook GA, Dalgleish A (2011). Infection, immunoregulation, and cancer. Immunol. Rev..

[21] Scudellari M (2017). News Feature: Cleaning up the hygiene hypothesis. Proc. Natl. Acad. Sci. USA.

[22] Cholapranee A, Ananthakrishnan AN (2016). Environmental Hygiene and Risk of Inflammatory Bowel Diseases: A Systematic Review and Meta-analysis. Inflamm. Bowel Dis..

[23] Lambrecht BN, Hammad H (2017). The immunology of the allergy epidemic and the hygiene hypothesis. Nat. Immunol..

[24] Olszak T (2012). Microbial exposure during early life has persistent effects on natural killer T cell function. Science.

[25] Kearney SC, Dziekiewicz M, Feleszko W (2015). Immunoregulatory and immunostimulatory responses of bacterial lysates in respiratory infections and asthma. Ann. Allergy Asthma Immunol..

[26] Zloza A (2018). Viruses, bacteria, and parasites - oh my! a resurgence of interest in microbial-based therapy for cancer. J. Immunother. Cancer..

[27] Kurtz J (2005). Specific memory within innate immune systems. Trends Immunol..

[28] Vivier E, Malissen B (2005). Innate and adaptive immunity: specificities and signaling hierarchies revisited. Nat. Immunol..

[29] Kaufmann E (2018). BCG Educates Hematopoietic Stem Cells to Generate Protective Innate Immunity against Tuberculosis. Cell.

[30] Kleinnijenhuis J (2012). Bacille Calmette-Guerin induces NOD2-dependent nonspecific protection from reinfection via epigenetic reprogramming of monocytes. Proc. Natl. Acad. Sci. USA.

[31] Naik S (2017). Inflammatory memory sensitizes skin epithelial stem cells to tissue damage. Nature.

[32] Ordovas-Montanes J (2018). Allergic inflammatory memory in human respiratory epithelial progenitor cells. Nature.

[33] Bazett M (2016). A novel microbe-based treatment that attenuates the inflammatory profile in a mouse model of allergic airway disease. Sci. Rep..

[34] Bazett M (2017). Attenuating immune pathology using a microbial-based intervention in a mouse model of cigarette smoke-induced lung inflammation. Respir. Res..

[35] Sham, H. P. *et al*. Immune Stimulation Using a Gut Microbe-Based Immunotherapy Reduces Disease Pathology and Improves Barrier Function in Ulcerative Colitis. *Frontiers in Immunology* **9** (2018). 10.3389/fimmu.2018.02211 PMC6170651 30319652

[36] Qu Biologics Inc. Safety and Efficacy of QBECO in Moderate to Severe Ulcerative Colitis. (2017).

[37] Sutcliffe S (2019). Novel Microbial-Based Immunotherapy Approach for Crohn’s Disease. Front. Med. (Lausanne).

[38] Yu YR (2016). A Protocol for the Comprehensive Flow Cytometric Analysis of Immune Cells in Normal and Inflamed Murine Non-Lymphoid Tissues. Plos One.

[39] Iizawa Y, Kitamoto N, Hiroe K, Nakao M (1996). Streptococcus pneumoniae in the nasal cavity of mice causes lower respiratory tract infection after airway obstruction. J. Med. Microbiol..

[40] Netea MG (2009). Circulating lipoproteins are a crucial component of host defense against invasive Salmonella typhimurium infection. Plos One.

[41] Loomis WP (2014). Temporal and anatomical host resistance to chronic Salmonella infection is quantitatively dictated by Nramp1 and influenced by host genetic background. Plos One.

[42] Siebenhaar F (2007). Control of Pseudomonas aeruginosa skin infections in mice is mast cell-dependent. Am. J. Pathol..

[43] King S, Metzger WI (1968). A new plating medium for the isolation of enteric pathogens. I. hektoen enteric agar. Appl. Microbiol..

