# Supplementary material for: Distinct inactivated bacterial-based immune modulators vary in their therapeutic efficacies for treating disease based on the organ site of pathology
Source: Sci Rep. 2020 Apr 3;10:5901. doi: 10.1038/s41598-020-62735-z (PMC7125210; doi:10.1038/s41598-020-62735-z)
Supplement: Supplementary file 1 — Supplementary Data. [file 41598_2020_62735_MOESM1_ESM.pdf]

**Supplementary Information for: Distinct inactivated bacterial-based immune modulators vary in their therapeutic efficacies for treating disease based on the organ site of pathology**

Authors: Shirin Kalyan[1,2&], Mark Bazett[1&], Ho Pan Sham[1], Momir Bosiljcic[1], Beryl Luk[1], Salim Dhanji[1], Amanda M. Costa[4], Stephanie WY Wong[1], Mihai G. Netea[3], David W. Mullins[4], Hal Gunn[1*]

[1]Qu Biologics Inc., Burnaby, BC, V5G 4X4, Canada; [2]Department of Medicine, University of British Columbia; [3]Department of Internal Medicine, Radboud University Medical Center, [4]Department of Microbiology and Immunology and Department of Medical Education, Geisel School of Medicine at Dartmouth, Hanover, NH 03755, USA

**Supplemental Figure 1.**

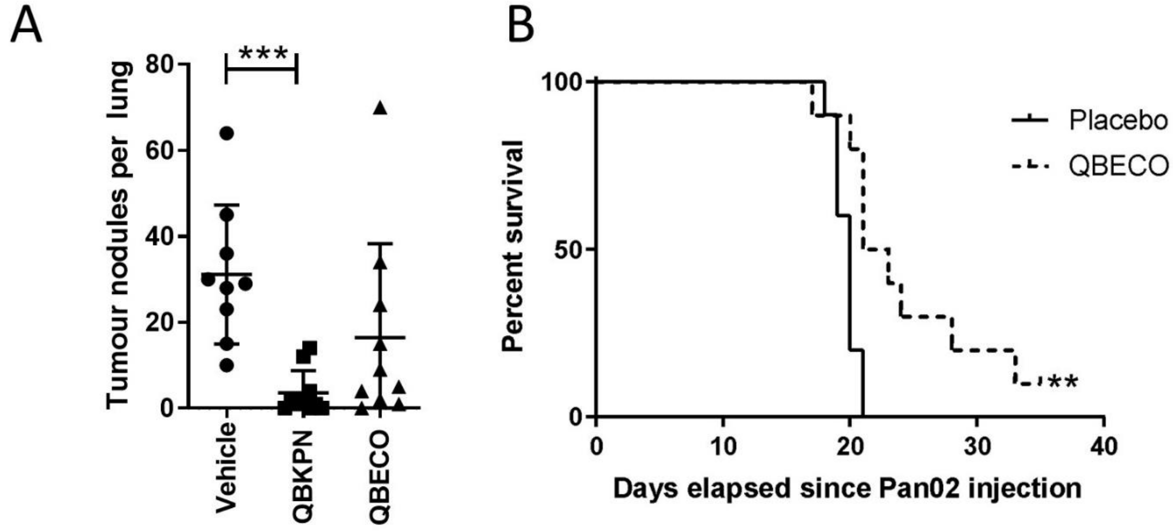


**Anti-cancer efficacy is organ specific, but not cancer type specific. (A)** Lung surface tumour count in C57BL/6 mice 17 days after seeding with B16F10 melanoma cells via tail vein injection, and administered vehicle, QBKPN and QBECO every second day by subcutaneous injection, starting 10 days before tumour seeding. N = 9-10 mice per group. Mean ± SD is shown, but statistical analysis was performed using non-parametric analysis to avoid making assumptions about distribution. *** $p < 0.001$ (Kruskal-Wallis test with Dunn's multiple comparisons). **(B)** Survival curve of mice seeded by interperitoneal injection with Panc02 pancreatic ductal adenocarcinoma cells. Vehicle or QBECO treatment was administered every second day by subcutaneous injection, starting 10 days before tumour seeding. N = 10 mice per group. ** $p < 0.01$, Log-rank test.

**Supplemental Figure 2.**

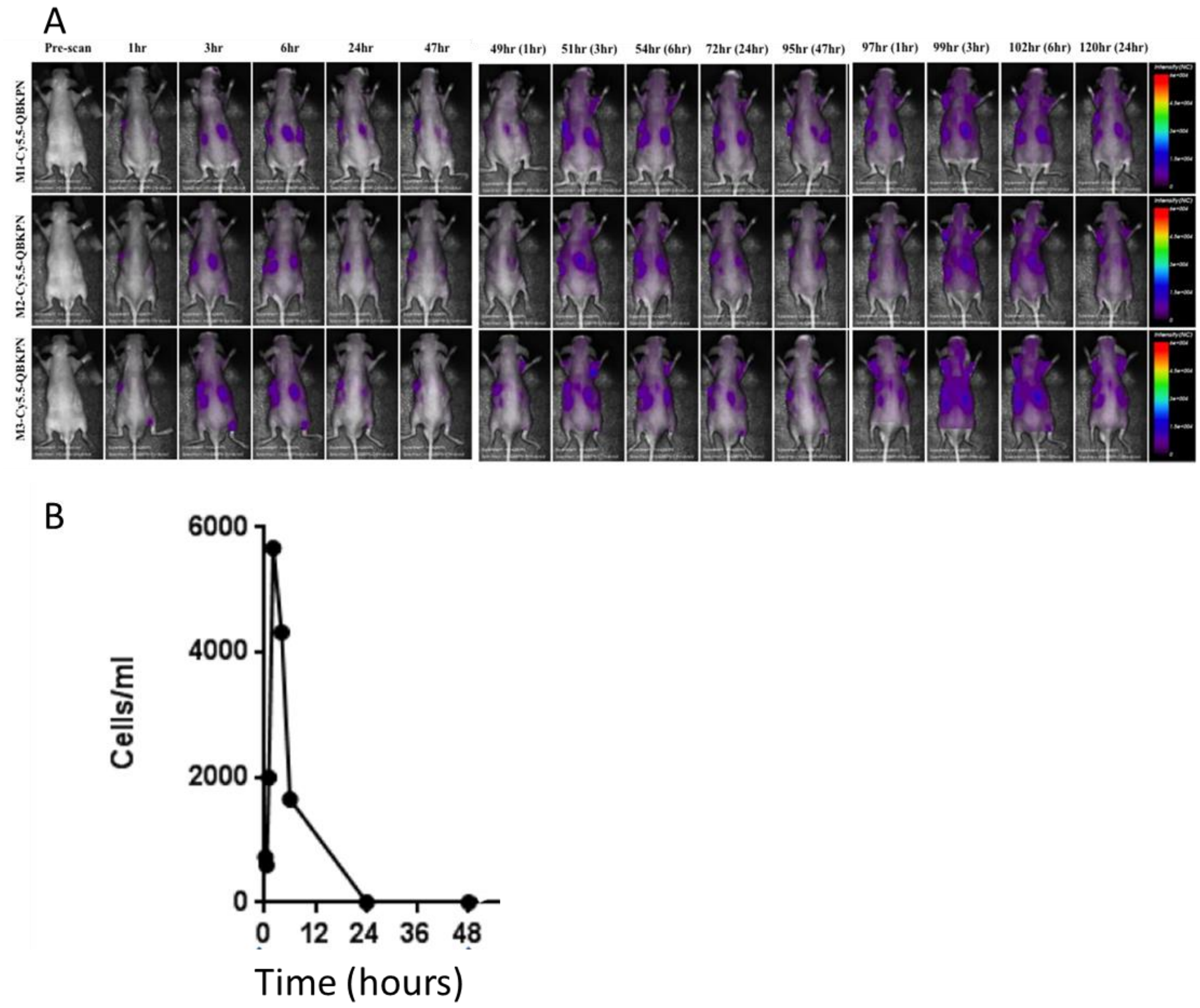


**Distribution of fluorescently labelled QBKPN after 3 subcutaneous injections. A** Fluorescent intensity maps of *in vivo* optical image of Cy5.5-QBKPN after 1, 2 or 3 subcutaneous injections. QBKPN was administered at 0 hours, 48 hours and 96 hours. N = 3 mice. **(B)** Pharmackinetic data depicting the presence of Cy5.5-labeled QBKPN (cells/mL) in blood after a single subcutaneous injection of Cy5.5-QBKPN. Blood was collected from three mice at rotating time points with each point representing the value from a single mouse.

**Supplemental Figure 3.**

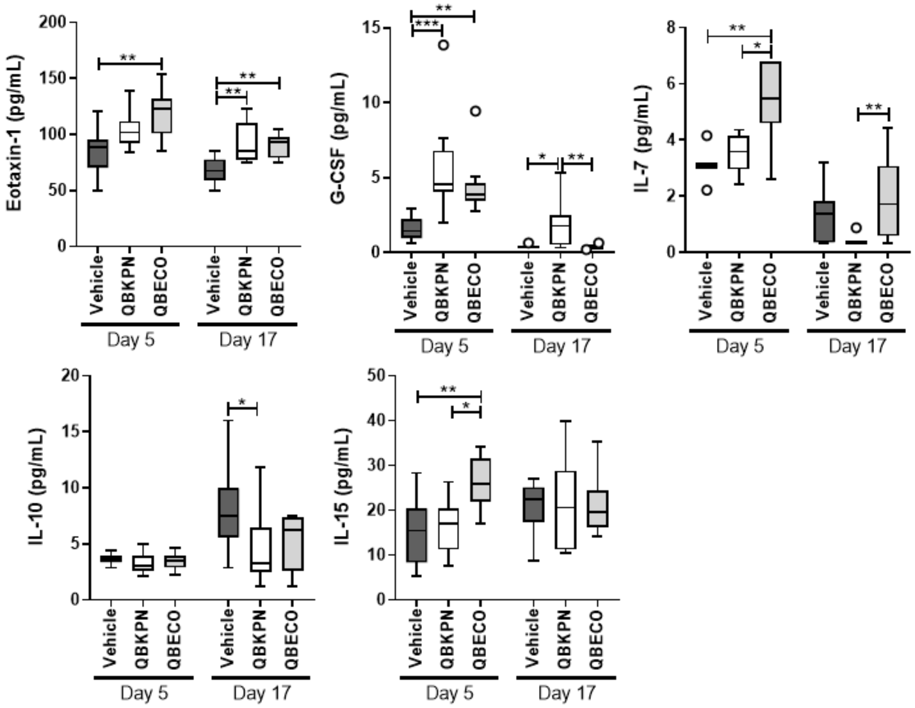


**Cytokine changes in the lung tissue in cancer bearing mice at day 5 and day 17 post-tumour inoculation.** Cytokines were measured in lung homogenates in the B16F10 lung cancer model at 5 and 17 days post tumour inoculation. Cytokines were measured by 31-cytoplex. GM-CSF (day 5), IL-1α, IL-1β, IL-2, IL-4, IL-6, IL-9, IL-12p40, IL-17, IFNγ, LIF, LIX, VEGF, CXCL1, M-CSF, MIP1α, MIP1β (day 5) and TNFα were detected but no significant changes were found between treatments. GM-CSF (day 17), IL-3, IL-5, IL12p70 and MIP-1β (day 17) were measured but were below the level of detection. Data are presented using Tukey boxplots. N = 8-10 mice per group. * $p < 0.05$; ** $p < 0.01$; *** $p < 0.001$, Kruskal-Wallis test with Dunn's multiple comparisons.

**Supplemental Figure 4.**

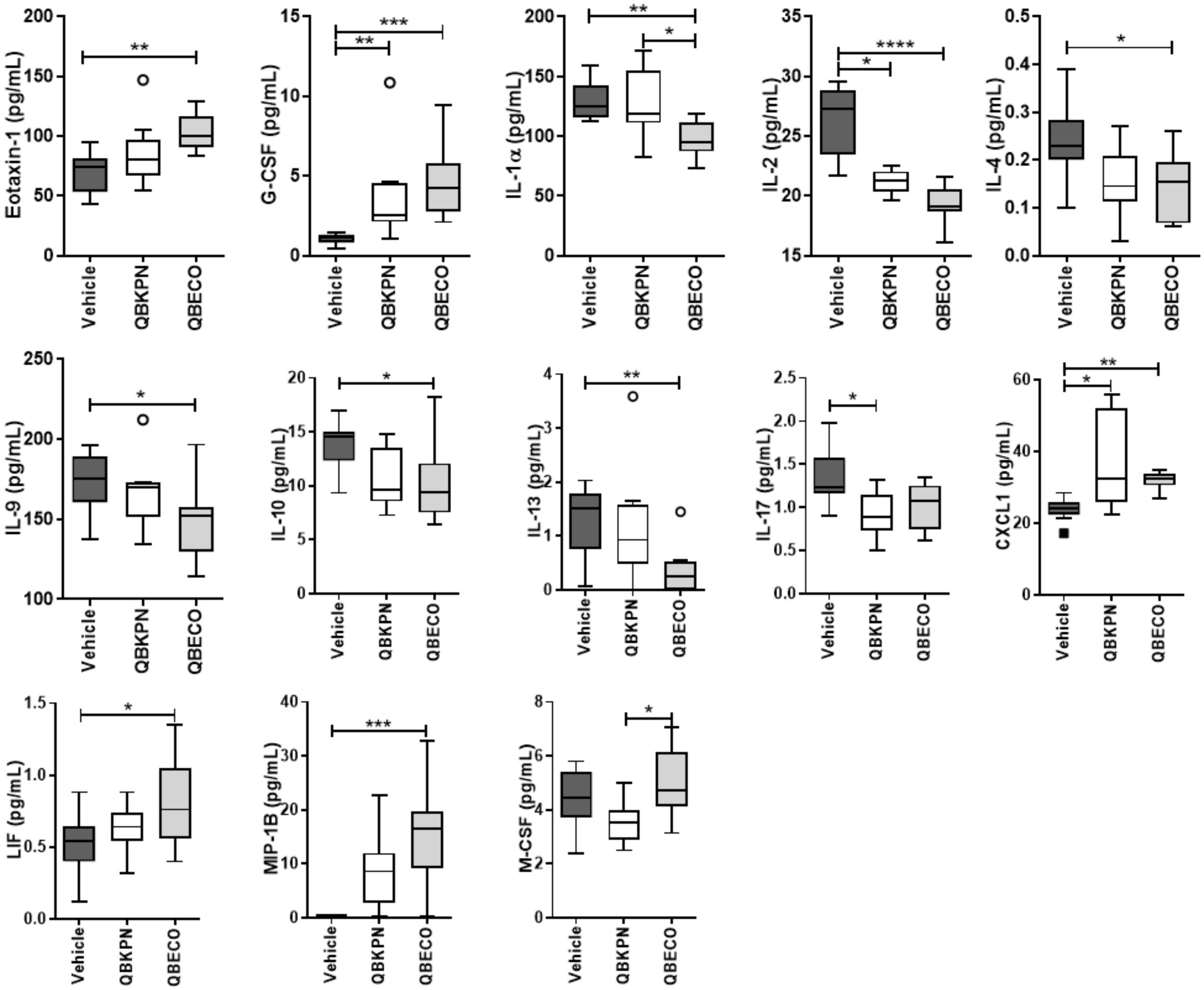


**Cytokine changes in the lung tissue in cancer free mice after 27 days of treatment.** Cytokines were measured in lung homogenates in cancer free mice at 27 days after initiating treatment with vehicle, QBKPN or QBECO, administered every-second day. Cytokines were measured by 31-cytoplex. GM-CSF, IFNγ, IL-1β, IL-6, IL-9, IL-7, IL-10, IL-15, LIX, TNFα, MIP1α, MIP1β and VEGF were detected, but there were no significant differences by Kruskal-Wallis analysis. IL-3, IL-5 and IL12p70 and MIP-1β measurement were below the level of detection. Data are presented using Tukey boxplots. N = 8-10 mice per group. * $p < 0.05$; ** $p < 0.01$; *** $p < 0.001$; **** $p < 0.0001$, Kruskal-Wallis test with Dunn's multiple comparisons.

**Supplemental Figure 5.**

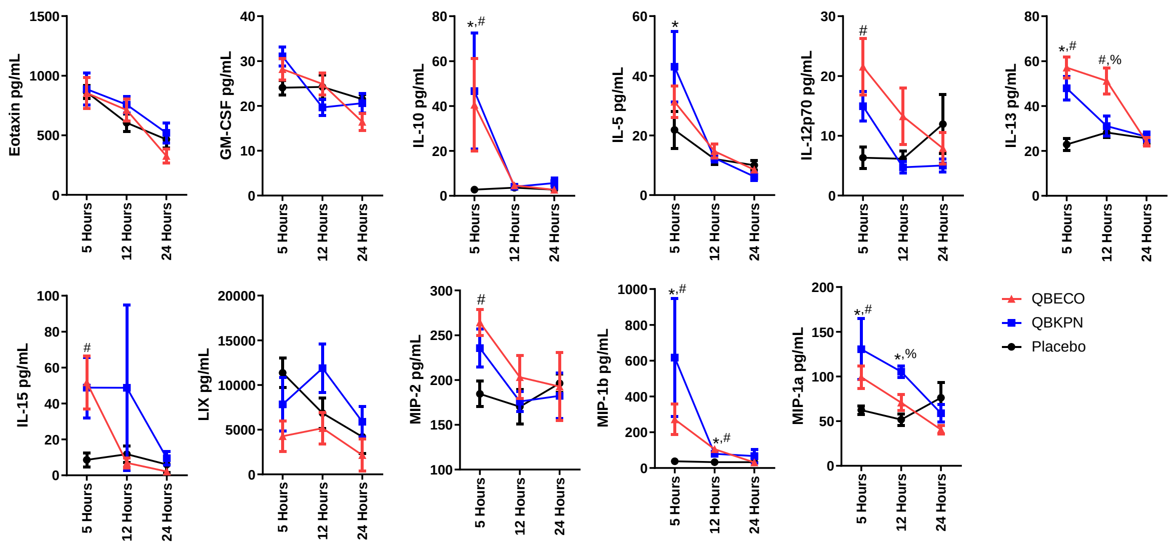


**Cytokine changes in the serum after a single infection of vehicle, QBKPN and QBECO.** Cytokines were measured in serum in cancer naïve C57BL/6 mice 5, 12 and 24 hours after a single subcutaneous injection of vehicle, QBKPN or QBECO. Cytokines were measured by 31-cytoplex. Eotaxin-1, IL-2, IL-1α, IL-7, IL-9, and VEGF were detected but no significant changes were found. IFNγ, IL-3, IL-4, and LIF measurement were below the level of detection. Data is depicted with the mean ± SD, but statistical analysis was performed using non-parametric analysis to avoid making assumptions about data being normally distributed. N = 8-10 mice per group. * $p < 0.05$ between QBKPN and vehicle; # $p < 0.05$ between QBECO and vehicle; * $p < 0.05$ between QBKPN and QBECO; Kruskal-Wallis test with Dunn's multiple comparisons. Data from the 5 hour data point comparing vehicle and QBKPN treated mice has been previously reported in https://www.tandfonline.com/doi/full/10.1080/2162402X.2017.1398875.

**Supplemental Figure 6.**

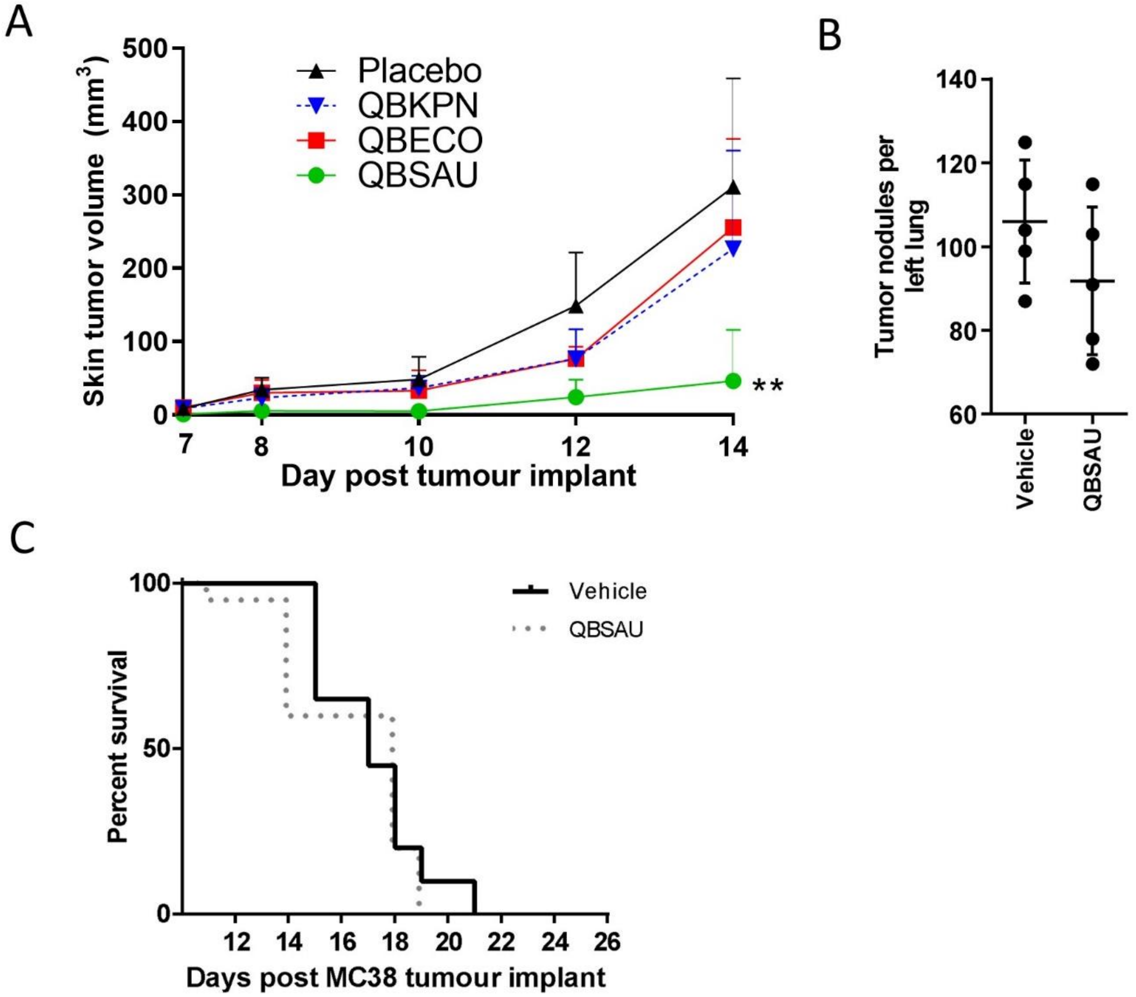


**QBSAU has anti-cancer efficacy in skin cancer models, but not in the lung and intraperitoneal cancer models. (A)** Skin tumor size in a mouse model where B16F10 cells are seeded dorsally via subcutaneous injection. Vehicle, QBKPN, QBECO and QBSAU administered by ventral subcutaneous injected every second day starting 10 days before tumour seeding and continued throughout the experiment. N = 5 per group. ** $p < 0.05$, repeated measures ANOVA. **(B)** Lung tumour count in the RFP-LLC mouse lung cancer model at day 15 mice administered with vehicle and QBSAU. Vehicle data reproduced from Fig. 1A. **(C)** Survival curve in the MC-38 mouse model in mice administered with vehicle and QBSAU. Vehicle data reproduced from Fig. 1B.

**Supplemental Figure 7.**

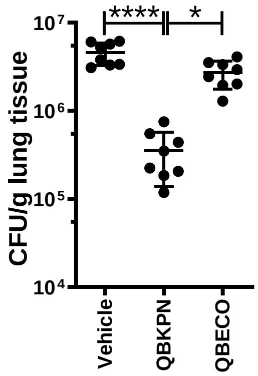


**Demonstration that organ-specific pathogen protection is independent of disease-causing pathogen.** *P. aeruginosa* count in the lungs of mice 3 days after bacterial challenge by intratracheal instillation. Mice were treated with vehicle, QBKPN or QBECO every second day starting 14 days before bacterial challenge. N = 8 mice per group. Data are shown with the mean ± SD, however, statistical analysis used non-parametric tests to avoid making assumptions about distributions. * $p < 0.05$; **** $p < 0.0001$, Kruskal-Wallis test with Dunn's multiple comparisons.
